# Decoupling the downstream effects of germline nuclear RNAi reveals that H3K9me3 is dispensable for heritable RNAi and the maintenance of endogenous siRNA-mediated transcriptional silencing in *Caenorhabditis elegans*

**DOI:** 10.1186/s13072-017-0114-8

**Published:** 2017-02-15

**Authors:** Natallia Kalinava, Julie Zhouli Ni, Kimberly Peterman, Esteban Chen, Sam Guoping Gu

**Affiliations:** 1 Department of Molecular Biology and Biochemistry, Rutgers the State University of New Jersey, Piscataway, NJ 08854 USA; 2 Nelson Labs A125, 604 Allison Road, Piscataway, NJ 08854 USA

## Abstract

**Background:**

Germline nuclear RNAi in *C. elegans* is a transgenerational gene-silencing pathway that leads to H3K9 trimethylation (H3K9me3) and transcriptional silencing at the target genes. H3K9me3 induced by either exogenous double-stranded RNA (dsRNA) or endogenous siRNA (endo-siRNA) is highly specific to the target loci and transgenerationally heritable. Despite these features, the role of H3K9me3 in siRNA-mediated transcriptional silencing and inheritance of the silencing state at native target genes is unclear. In this study, we took combined genetic and whole-genome approaches to address this question.

**Results:**

Here we demonstrate that siRNA-mediated H3K9me3 requires combined activities of three H3K9 histone methyltransferases: MET-2, SET-25, and SET-32. *set*-*32* single, *met*-*2 set*-*25* double, and *met*-*2 set*-*25;set*-*32* triple mutant adult animals all exhibit prominent reductions in H3K9me3 throughout the genome, with *met*-*2 set*-*25;set*-*32* mutant worms losing all detectable H3K9me3 signals. Surprisingly, loss of high-magnitude H3K9me3 at the native nuclear RNAi targets has no effect on the transcriptional silencing state. In addition, the exogenous dsRNA-induced transcriptional silencing and heritable RNAi at *oma*-*1*, a well-established nuclear RNAi reporter gene, are completely resistant to the loss of H3K9me3.

**Conclusions:**

Nuclear RNAi-mediated H3K9me3 in *C. elegans* requires multiple histone methyltransferases, including MET-2, SET-25, and SET-32. H3K9me3 is not essential for dsRNA-induced heritable RNAi or the maintenance of endo-siRNA-mediated transcriptional silencing in *C. elegans*. We propose that siRNA-mediated transcriptional silencing in *C. elegans* can be maintained by an H3K9me3-independent mechanism.

**Electronic supplementary material:**

The online version of this article (doi:10.1186/s13072-017-0114-8) contains supplementary material, which is available to authorized users.

## Background

Following the initial discovery of RNAi [1, 2], a variety of small RNA-mediated silencing phenomena have been uncovered. There is a considerable diversity in the biogenesis of small RNA, biochemical function of the Argonaute (AGO) protein, as well as downstream effects among different silencing pathways that involve small RNA. In addition to the posttranscriptional gene silencing (PTGS) mechanism, in which the AGO-siRNA complexes, also referred to as RNA-induced silencing complex (RISC), degrade target mRNA [3–6], RNAi can also induce heterochromatin and (co-)transcriptional gene silencing at the target locus (reviewed in [7–11]). These so-called nuclear RNAi effects, initially discovered in plants and *Schizosaccharomyces pombe*, provide one of the first examples that RNA can function as a sequence-specific guide to regulate chromatin structure. Subsequent studies in these systems revealed that nuclear RNAi plays critical roles in gene regulation, heterochromatin assembly, genome surveillance and chromosome stability. Recent studies have shown that the nuclear RNAi pathway and its role in genome surveillance are conserved in animals as well. In *Drosophila melanogaster*, PIWI protein and the PIWI-interacting RNA (piRNA) silence transposons through both PTGS and transcriptional gene silencing (TGS) mechanisms [12–15]. piRNA-dependent heterochromatin formation at transposable elements was also shown to occur in mammalian germ cells [16].

In *Caenorhabditis elegans,* nuclear RNAi is required for H3K9me3 and transcriptional silencing in a distinct set of genomic loci that have high levels expression of endo-siRNA (more on the native targets later in Background). Besides endo-siRNA, exogenous dsRNA can also trigger highly specific nuclear RNAi effects at native genes or transgenes [17–21]. The dsRNA-induced H3K9me3 in *C. elegans* can last for at least three generations after the initial dsRNA exposure has been removed [18].

Several NRDE (nuclear RNAi-defective) proteins [19, 20, 22] and a germline nuclear Argonaute protein, HRDE-1 [17, 21, 23], are essential for nuclear RNAi in *C. elegans*, but not PTGS. Previous studies have indicated that dsRNA-triggered gene silencing can persist for multiple generations in *C. elegans* [24–26]. Mutant *C. elegans* strains lacking nuclear RNAi components (e.g., HRDE-1, NRDE-1, or NRDE-2) are defective in heritable RNAi induced by either dsRNA or piRNA [17, 21, 23, 24] and exhibit other transgenerational defects, such as the mortal germline (Mrt) phenotype [17, 21] and heat-induced progressive activation of native target genes [27]. These features make *C. elegans* a uniquely attractive system to study the mechanisms of RNA-mediated chromatin regulation and transgenerational epigenetics, as well as their roles in germline development.

Methylation of histone H3 at lysine 9 (H3K9me), the hallmark of constitutive heterochromatin, is an evolutionarily conserved response of nuclear RNAi [9, 28]. Studies in *S. pombe* have indicated a complex role of H3K9me2/3. Tethering H3K9 methyltransferase (HMT), Clr4, to a target gene leads to transcriptional silencing [29, 30]. H3K9 methylation is also required for stable interaction between RNAi machineries and chromatin [9], which convolutes the determination of the direct cause of transcriptional silencing—whether it being heterochromatin, RNAi, or both. The complexity of the system is further evidenced by the role of heterochromatin in promoting co-transcriptional silencing [31].

In *C. elegans*, H3K9me3-marked regions form large domains, enriched in the arms of autosomes, particularly the ones associated with meiotic pairing center, and the left tip of the sex chromosome [32, 33]. Previous studies have shown that H3K9 methylation is involved in a diverse set of functions: germline development and immortality [12, 34–36], chromosome nuclear localization [37], dosage compensation [38], RNAi [18, 21, 22, 36], and maintaining the genome stability [39].

We previously showed that germline nuclear RNAi-dependent H3K9me3 is highly enriched in long terminal repeat (LTR) retrotransposons [40]. Even though these regions account for only a small fraction of the total H3K9me3-enriched regions in *C. elegans* genome, H3K9me3 profiles at these native germline nuclear RNAi targets are prominent and defined [17, 40]. By performing whole-genome analyses using the *hrde*-*1* loss-of-function mutant, we identified loci with the *g*ermline nuclear RNAi-dependent heterochromatin (GRH) and loci with *g*ermline nuclear RNAi-dependent transcriptional silencing (GRTS) in the *C. elegans* genome [40]. Interestingly, GRTS and GRH loci only partially overlap. GRTS loci tend to have much less H3K9me3 defects than the GRH loci in *hrde*-*1* mutants. Conversely, many GRH loci show little changes in transcriptional repression in *hrde*-*1* mutants. These results highlight the complexity of germline nuclear RNAi in *C. elegans,* suggesting that the two germline nuclear RNAi effects, H3K9me3 and transcriptional silencing, may not be causally linked.

In this study, we combined genetic and whole-genome approaches to characterize the requirement of H3K9me3 for transcriptional silencing at nuclear RNAi targets. *C. elegans* has 38 putative histone methyltransferases (HMTs) [41, 42]. It is unclear which of them are required for the H3K9me3 response associated with nuclear RNAi. MET-2 (a H3K9 mono- and dimethylation HMT) [43] and SET-25 (a H3K9 trimethylation HMT) are required for all detectable H3K9me3 at the embryonic stage, as shown by mass spectrometry analysis [37, 44]. A recent study also showed a complete loss of H3K9me3 in adult germline of *met*-*2 set*-*25* mutants by immunofluorescence (IF) analysis and de-silencing in the *met*-*2 set*-*25* mutants leads to increased genome-instability and mutation [39]. Interestingly, many H3K9me3-enriched loci, including LTR retrotransposons, remain silenced in *met*-*2 set*-*25* mutant worms [39]. Despite the prominent loss of H3K9me3 in *met*-*2 set*-*25* mutants, SET-32 and SET-26 were also shown to be H3K9 HMTs by IF [38] and in vitro HMT assay [42], respectively. A candidate screen using a transgene reporter showed that SET-25 is required for exogenous dsRNA-induced heritable RNAi and SET-32 is required for piRNA-induced gene silencing [21]. The H3K9me3 status at these nuclear RNAi reporter transgenes is unclear. In addition, the requirement of H3K9me3 for nuclear RNAi-mediated transcriptional silencing at native genes has not been tested.

## Results

### MET-2, SET-25, and SET-32 are required for exogenous dsRNA-triggered H3K9me3

We first performed H3K9me3 ChIP-seq in *met*-*2 set*-*25* double mutant worms to examine the requirement of these two HMTs for exogenous dsRNA-triggered H3K9me3. We chose a germline-specific gene *oma*-*1* as the target gene as it is sensitive to dsRNA-induced nuclear RNAi [17, 45]. Despite the popular usage of *oma*-*1* in nuclear RNAi and heritable silencing studies (e.g., [17, 24, 45, 46]), a high-resolution profile of dsRNA-induced H3K9me3 at this locus has not been reported before. A combination of control samples, including wild-type and *hrde*-*1* mutant animals, both with *oma*-*1* RNAi, as well as wild-type animals with control RNAi (*gfp*), were used to indicate the full extent of HRDE-1-dependent H3K9me3 response at the *oma*-*1* locus. Synchronized young adult animals, of which over 50% of total cells are germline [47], were used throughout this study. (Additional file 1: Table S1 lists all samples and sequencing libraries used in this study.)

**Fig. 1:**
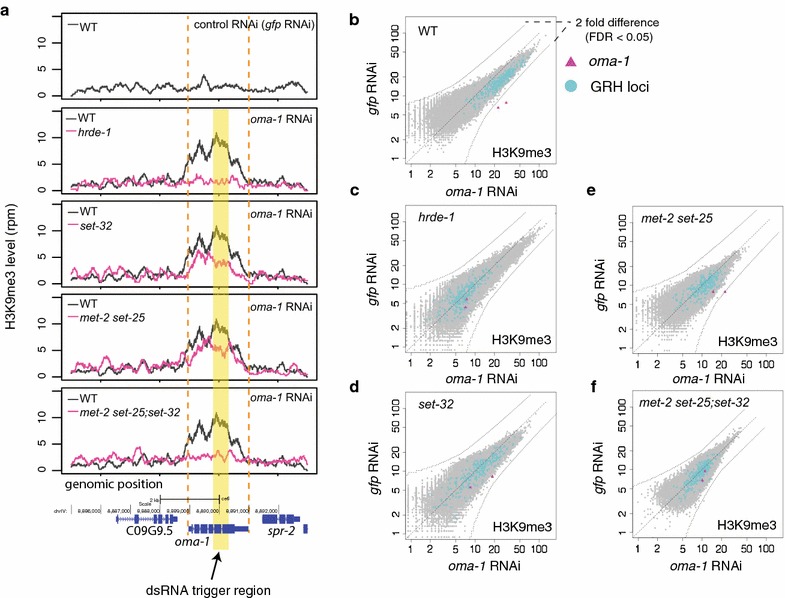
MET-2, SET-25, and SET-32 are required for the dsRNA-triggered H3K9me3 response at *oma*-*1.* **a** H3K9me3 levels in different samples are plotted as a function of position along the *oma*-*1* locus. The WT response (*oma*-*1* RNAi) is shown in all panels, except the *top one* (WT with *gfp* RNAi), to facilitate comparison with mutant ones. A *yellow block* indicates the region targeted by dsRNA. **b**–**f** Scatter plots that compare whole-genome H3K9me3 levels in *oma*-*1* RNAi and *gfp* RNAi samples at 1 kb resolution for WT and various mutant strains. *oma*-*1* regions (2 kb) and GRH loci (215 kb) are *highlighted*. *Curved dotted lines* indicate the twofold change (FDR < 0.05). Synchronized young adult animals (19 °C) were used throughout this study

The *oma*-*1* dsRNA-induced H3K9me3 response was analyzed using coverage plot (Fig. 1a) and whole-genome 1-kb-resolution scatter plot (Fig. 1b–f). In WT, *oma*-*1* dsRNA-induced H3K9me3 peaked at the dsRNA trigger region (0.52 kb) (Fig. 1a) and was limited to *oma*-*1* (Fig. 1b), a degree of specificity similar to the ones observed for other targets in our previous study [40]. The H3K9me3 response spread throughout the *oma*-*1* gene (~2 kb) and dropped to the background level around the putative *oma*-*1* promoter and the polyadenylation site, without spreading into either of the adjacent genes, both of which are less than 0.5 kb away (Fig. 1a). The H3K9me3 response was absent in the *hrde*-*1* mutant (*oma*-*1* RNAi) or WT (control RNAi) worms (Fig. 1a–c), as expected [17, 45].

Compared to WT, *met*-*2 set*-*25* mutants showed only a partial defect in the H3K9me3 response at the *oma*-*1* locus (Fig. 1a–e), suggesting that additional H3K9 HMT(s) are involved. This was somewhat surprising because of the more severe H3K9me3 defect at the whole-genome level observed in the same mutant sample (Fig. 4a and see later section). In addition, previous studies showed that *met*-*2 set*-*25* double mutants were devoid of H3K9me3 at the embryo stage [37, 44], as well as in adult germline [39].

We then performed *oma*-*1* RNAi and H3K9me3 ChIP-qPCR to screen additional H3K9 HMT mutants (Additional file 2: Fig. S1), among which only *set*-*32* mutant and *met*-*2 set*-*25;set*-*32* triple mutant worms showed defects in the H3K9me3 response at the *oma*-*1* locus (Additional file 2: Fig. S1). *met*-*2* or *set*-*25* single mutants did not show any defect in the H3K9me3 response (Additional file 2: Fig. S1). We further confirmed the requirement of *set*-*32* by H3K9me3 ChIP-seq analysis (Fig. 1a). Compared to *met*-*2 set*-*25* mutants (*oma*-*1* RNAi), *set*-*32* mutants (*oma*-*1* RNAi) showed a stronger defect in the H3K9me3 response at the *oma*-*1* locus (∆H3K9me3_*oma*-*1*_ [*set*-*32*/WT] = 0.32 and ∆H3K9me3_*oma*-*1*_ [*met*-*2 set*-*25*/WT] = 0.55) (Fig. 1a, d, e), indicating that SET-32 plays a more prominent role than MET-2 SET-25 in dsRNA-induced H3K9me3. Similar to *hrde*-*1* mutants (*oma*-*1* RNAi), *met*-*2 set*-*25;set*-*32* mutants (*oma*-*1* RNAi) showed a background level of H3K9me3 ChIP-seq signal at the *oma*-*1* locus (Fig. 1a, c, f), suggesting that MET-2, SET-25, and SET-32, in combination, contribute to the full H3K9me3 response induced by exogenous dsRNA.

### H3K9me3 is not required for exogenous dsRNA-induced transcriptional silencing and heritable RNAi

**Fig. 2:**
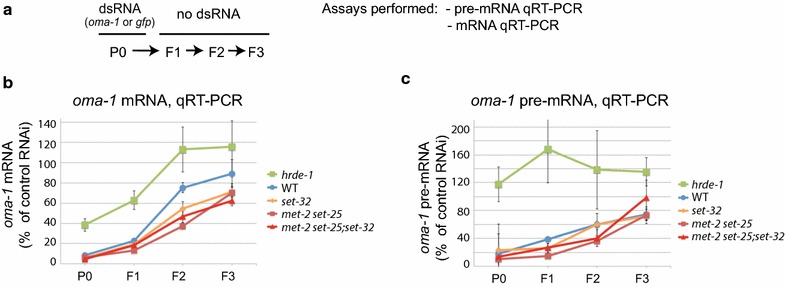
Requirement of H3K9me3 in heritable RNAi and transcriptional silencing of *oma*-*1*. **a** Experimental scheme. **b**, **c** mRNA and pre-mRNA levels of *oma*-*1* RNAi samples, normalized to the control RNAi, are plotted as a function of generations for WT or various mutant strains

To investigate the role of H3K9me3 in nuclear RNAi and heritable silencing, we performed a set of heritable RNAi experiments using wild-type, *hrde*-*1, met*-*2 set*-*25, set*-*32,* and *met*-*2 set*-*25;set*-*32* mutant strains with *oma*-*1* or *gfp* RNAi (Fig. 2a). qRT-PCR analyses of *oma*-*1* mRNA and pre-mRNA were performed for the dsRNA-exposed animals (P0 generation) and their descendants (F1, F2, and F3) that were cultured without dsRNA exposure.

In the WT animals, *oma*-*1* RNAi caused heritable silencing of the target gene in F1, F2, and F3 at both mRNA and pre-mRNA levels (Fig. 2b, c). The heritable silencing was dependent on HRDE-1 (Fig. 2b, c), as expected [17, 24].

All three HMT mutant strains (*met*-*2 set*-*25, set*-*32, and met*-*2 set*-*25;set*-*32*) showed wild-type-like multigenerational profiles of *oma*-*1* pre-mRNA (Fig. 2c), despite various degrees of H3K9me3 defect at the *oma*-*1* locus. These results indicate that heritable transcriptional silencing can occur in the absence of the H3K9me3 response. We note that all three HMT mutant strains had a modest but consistently enhanced heritable RNAi at the mRNA level (Fig. 2b).

**Fig. 3:**
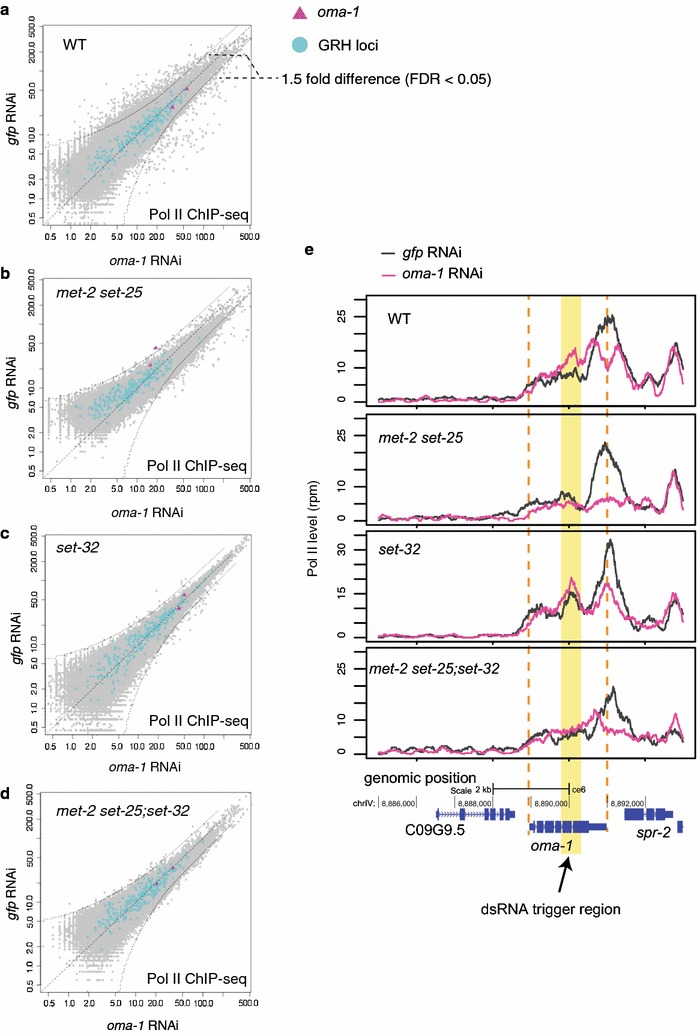
Impact of RNAi on Pol II profile at the *oma*-*1* locus. **a**–**d** Scatter plots that compare whole-genome Pol II levels in *oma*-*1* RNAi and *gfp* RNAi samples at 1 kb resolution for WT and various mutant strains. *oma*-*1* regions (2 kb) and GRH loci (215 kb) are *highlighted*. *Curved dotted lines* indicate the 1.5-fold change (FDR < 0.05). **e** Pol II levels are plotted as a function of position along the *oma*-*1* locus

To further examine nuclear RNAi-mediated transcriptional silencing at *oma*-*1*, we performed RNA polymerase II (Pol II) ChIP-seq for the P0 generation samples, using an antibody against the phosphorylated C-terminal domain (CTD) repeat YSPTSPS at the S2 position (S2P), a modification associated with the elongating Pol II. Our scatter plot analysis showed that, compared to *gfp* RNAi, *oma*-*1* RNAi did not change the overall Pol II levels at the *oma*-*1* locus in wild-type, *set*-*32,* or *met*-*2 set*-*25;set*-*32* mutant worms (Fig. 3a, c, d) [a modest reduction in the Pol II level was observed in the *oma*-*1* RNAi sample for *met*-*2 set*-*25* mutants (Fig. 3b)]. However, coverage plot analysis showed that *oma*-*1* RNAi changed the Pol II profile at the target gene. Specifically, it led to a reduction in the Pol II level at the 3′ end (for WT and all three HMT mutant strains) and an increase in the gene body (all samples except *met*-*2 set*-*25* mutants) (Fig. 3e). Such Pol II shift is consistent with a model in which exogenous dsRNA-induced transcriptional silencing occurs at the elongation step, as previously suggested [19].

Taken together, these results indicate that MET-2, SET-25, and SET-32 in combination are responsible for the full H3K9me3 response for exogenous dsRNA-triggered H3K9me3. However, these HMTs are not required for dsRNA-induced transcription silencing or heritable silencing.

### MET-2, SET-25, and SET-32 in combination contribute to all of the detectable H3K9me3 at the native nuclear RNAi targets in adult animals

**Fig. 4:**
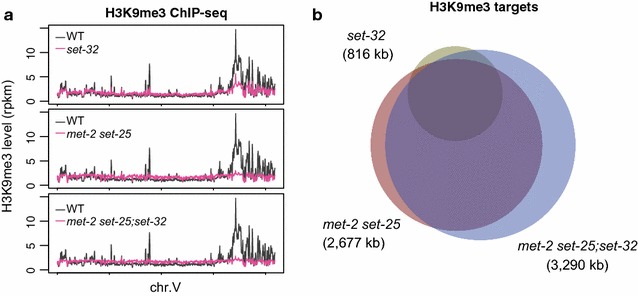
Global H3K9me3 contribution of MET-2, SET-25, and SET-32. **a** H3K9me3 levels are plotted as a function of position in chromosome V for WT and HMT mutant strains (the effect is the same for other chromosomes, see Additional file 3: Fig. S2). A moving average with a sliding window of 50 kb was used to plot H3K9me3 levels. **b** A Venn diagram of H3K9me3 regions that are dependent on SET-25, MET-2 SET-25, and MET-2 SET-25;SET-32. Targets were identified as regions with ∆H3K9me3[mutant/WT] ≤0.5 (FDR < 0.05) in two replica (Additional file 6: Fig. S3)

**Fig. 5:**
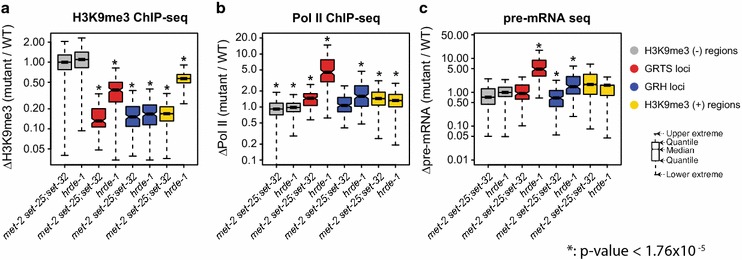
Impact of *met*-*2 set*-*25;set*-*32* mutations and *hrde*-*1* mutation on **a** H3K9me3, **b** Pol II, and **c** pre-mRNA in different subsets of the genome: GRTS loci (191 kb), GRH loci (215 kb), top 5-percentile H3K9me3 regions in WT [H3K9me3(+) regions, 4775 kb], and bottom 5 to 25-percentile H3K9me3 regions in WT [H3K9me3(−) regions, 20,200 kb]. Boxplot analysis is used to describe the ratios between mutant and WT for H3K9me3, Pol II, and pre-mRNA

When examined at the global level, *set*-*32, met*-*2 set*-*25*, and *met*-*2 set*-*25;set*-*32* mutant worms all showed significant reduction in the H3K9me3 ChIP-seq signal compared to WT (Fig. 4a and Additional file 3: Fig. S2). *met*-*2 set*-*25;set*-*32* triple mutants had the most severe global H3K9me3 loss among the three HMT mutant strains and showed H3K9me3 depletion throughout each of the six chromosomes (Fig. 4a and Additional file 3: Fig. S2). *met*-*2 set*-*25* double mutants ranked the second and had a severity of H3K9me3 loss similar to *met*-*2 set*-*25;set*-*32* mutants. *set*-*32* mutants showed the least global H3K9me3 loss among the three HMT mutant strains. To quantify the loss of H3K9me3, we defined the top 5 percentile regions of H3K9me3 in WT as the H3K9me3(+) regions (Additional file 4: Table S3). The median values of ∆H3K9me3[mutant/WT] for these H3K9me3(+) regions were 0.30, 0.25, and 0.17 for *set*-*32*, *met*-*2;set*-*25*, and *met*-*2;set*-*25;set*-*32* mutants, respectively (Fig. 5a and Additional file 6: Fig. S3a, all *p* values <2.2 × 10^−16^).

By using ∆H3K9me3[mutant/WT] ≤0.5 (FDR < 0.05) as the cutoff, we identified 816 kb regions with SET-32-dependent H3K9me3, which were much less than the ones that required MET-2 SET-25 (2677 kb) or all three HMTs (3290 kb) (Fig. 4b, Additional file 5: Fig. S4a, b, and Additional file 4: Table S3). Regions with SET-32-dependent H3K9me3 largely overlapped with the MET-2 SET-25-dependent ones (Fig. 4b). Taken together, these results indicate that MET-2, SET-25, and SET-32, in combination, contribute to all detectable level of H3K9me3 ChIP-seq signal in adult *C. elegans*. Interestingly, both *set*-*32* and *met*-*2 set*-*25* mutants had more than 50% H3K9me3 loss in many regions throughout the genome compared to wild-type animals, suggesting a synergistic relationship between SET-32 and MET-2 SET-25.

**Fig. 6:**
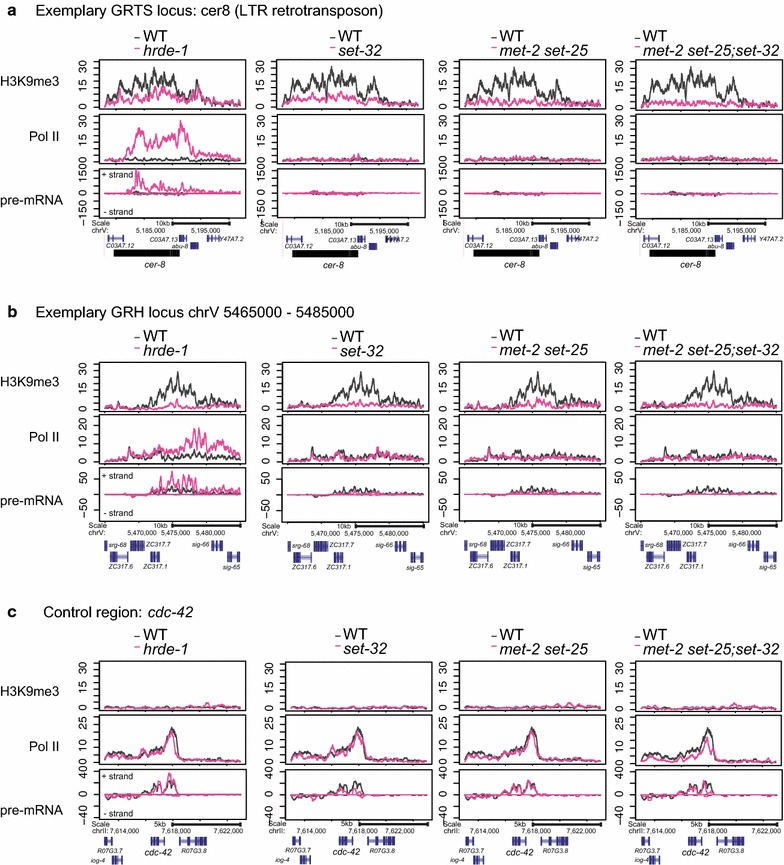
Coverage plots of H3K9me3, Pol II, and pre-mRNA in **a** an exemplary GRTS locus, **b** an exemplary GRH locus, and **c** a control region. For pre-mRNA coverage plots, sense reads are plotted above the *y* = 0 line (antisense bellow)

We then limited our analysis to the native targets of germline nuclear RNAi. Consistent with our previous work [40], *hrde*-*1* mutation led to a much stronger loss of H3K9me3 in GRH than GRTS loci, as shown in exemplary targets in Fig. 6a, b. Similar results were obtained when GRTS and GRH loci were analyzed as groups: the median values of ∆H3K9me3[*hrde*-*1*/WT] were 0.17 for GRH and 0.38 for GRTS loci (both *p* values <2.2 × 10^−16^, Fig. 5a and Additional file 6: Fig. S3a). The partial H3K9me3 loss in *hrde*-*1* mutants indicates that GRTS carry both HRDE-1-dependent and HRDE-1-independent H3K9me3.

*met*-*2 set*-*25;set*-*32* triple mutants showed a background level of H3K9me3 at both the GRTS and GRH nuclear RNAi targets (Fig. 6a, b). This is expected because of the aforementioned genome-wide depletion of H3K9me3 in the same mutant worms. *met*-*2 set*-*25* double mutant and *set*-*32* mutant worms both showed strong H3K9me3 loss at both GRTS and GRH loci (Fig. 6a, b). The median values of ∆H3K9me3[mutant/WT] for *set*-*32, met*-*2 set*-*25,* and *met*-*2 set*-*25;set*-*32* mutants were 0.21, 0.25 and 0.15 in GRH loci (0.23, 0.20, and 0.13 in the GRTS loci, respectively) (Fig. 5a and Additional file 6: Fig. S3a, all *p* values <2.2 × 10^−16^). Therefore, these HMTs together are required for HRDE-1-dependent and HRDE-1-independent H3K9me3 at the native germline nuclear RNAi targets.

### H3K9me3 is dispensable for transcriptional silencing at the native nuclear RNAi targets

To determine the impact of H3K9me3 loss on the transcriptional silencing in the native germline nuclear RNAi targets, we performed coverage analyses of Pol II ChIP-seq, and pre-mRNA-seq for two exemplary native targets and a control region (Fig. 6a–c). *set*-*32, met*-*2 set*-*25, and met2 set*-*25;set*-*32* mutants all showed background levels of Pol II occupancy and pre-mRNA at these two targets, similar to WT, despite partial or complete H3K9me3 loss in the mutants. In contrast, *hrde*-*1* mutant worms showed dramatic increases in both Pol II occupancy and pre-mRNA at these two targets, even though it had only partial H3K9me3 loss at *cer8,* a GRTS locus (Fig. 6a).

Consistent with our previous study, *hrde*-*1* mutant worms showed strong transcriptional de-silencing in GRTS loci: the median values of ∆Pol II [*hrde*-*1*/WT] and ∆pre-mRNA [*hrde*-*1*/WT] were 4.52 and 4.85, respectively (*p* values <3.42 × 10^−15^) (Fig. 5b, c). In contrast, *met*-*2 set*-*25;set*-*32* mutants showed only a modest increase in Pol II occupancy at GRH loci overall: the median value of ∆Pol II [*met2 set*-*25;set*-*32*/WT] was 1.46 (*p* value <3.01 × 10^−13^). Importantly, *met*-*2 set*-*25;set*-*32* mutants did not show any increase in pre-mRNA at GRTS loci: the median value of ∆pre-mRNA [*met2 set*-*25;set*-*32*/WT] was 0.93 (*p* value = 0.062). In addition, GRH loci showed unchanged Pol II occupancy in *met*-*2 set*-*25;set*-*32* mutants (the median value of ∆Pol II [*met2 set*-*25;set*-*32*/WT] = 1.07, *p* value = 0.16) and an unexpected decrease in pre-mRNA (the median value of ∆pre-mRNA [*met2 set*-*25;set*-*32*/WT] was 0.68, *p* value = 7.59 × 10^−10^) (Fig. 5b, c and Additional file 6: Fig. S3b-c).

We note that the H3K9me3(+) regions in *met*-*2 set*-*25;set*-*32* mutants, on average, showed a modest increase in the Pol II level over WT: the median value of ∆Pol II [*met2 set*-*25;set*-*32*/WT] was 1.44 (*p* value = 1.47 × 10^−11^) (Fig. 5b, c and Additional file 6: Fig. S3b, c). However, the pre-mRNA increase in these regions was not statistically significant: the median value of ∆pre-mRNA [*met2 set*-*25;set*-*32*/WT] was 1.70 (*p* value = 0.63). Scatter plot analyses of H3K9me3 ChIP-seq, Pol II ChIP-seq, pre-mRNA-seq, and mRNA-seq confirmed that the loss of H3K9me3 was not associated with transcriptional de-silencing in most of the H3K9me3(+) regions (Additional file 7: Fig. S5). Furthermore, the overall germline chromosome morphology was similar between *met*-*2 set*-*25;set*-*32* mutant and wild-type worms (Additional file 8: Fig. S6). These data suggest that H3K9me3 plays, at most, a very limited role in germline chromatin condensation or transcription silencing at the global level.

### Germline nuclear RNAi-mediated H3K9me3 is accompanied with H3K27me at the native targets

**Fig. 7:**
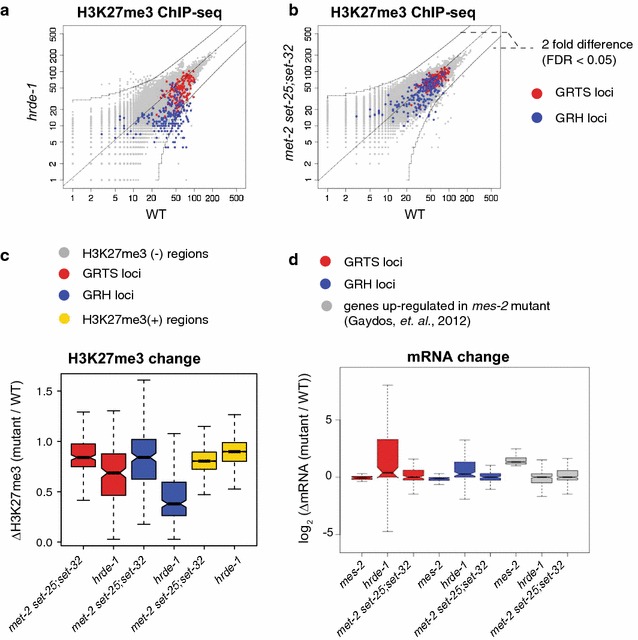
H3K27me3 is associated with native germline nuclear RNAi targets and not affected in *met*-*2 set*-*25;set*-*32* mutant worms. **a**, **b** Scatter plots comparing whole-genome H3K27me3 levels in WT and *hrde*-*1* or *met*-*2 set*-*25;set*-*32* mutants at 1 kb resolution. **c** Boxplot analysis showing H3K27me3 changes between WT and *met*-*2 set*-*25;set*-*32* or *hrde*-*1* mutant worms in different subsets of the genome: GRTS loci (191 kb), GRH loci (215 kb), top 5-percentile H3K27me3 regions in WT (H3K27me3 + regions, 4966 kb). **d** Boxplot analysis comparing mRNA expression between WT and *mes*-*2, hrde*-*1, or met*-*2 set*-*25;set*-*32* mutants in different subsets of the genome: GRTS loci (121 genes), GRH loci (142 genes), genes that are up-regulated in *mes*-*2* mutant worms (355 genes, ≥2 fold, FDR < 0.05). Published microarray data [48] were used for mRNA expression changes between *mes*-*2* mutant and WT worms

To characterize the other known germline nuclear RNAi-mediated heterochromatin mark in *C. elegans*, H3K27me3 [45], at the whole-genome level, we performed H3K27me3 ChIP-seq in the wild-type and *hrde*-*1* mutant worms. Consistent with a previous study [45], we found that H3K27me3 was associated with both GRH and GRTS loci and was dependent on HRDE-1 (Fig. 7a, c). The median values for ∆H3K27me3 [*hrde*-*1/*WT] at GRTS and GRH loci were 0.68 and 0.38, respectively (all *p* values <2.2 × 10^−16^).

Just like H3K9me3, the correlation between HRDE-1-dependent-H3K27me3 and transcriptional silencing was different between the GRTS and GRH loci: GRTS loci showed a much weaker H3K27me3 loss than GRH loci in *hrde*-*1* mutants (Fig. 7a, c), comparing with a much stronger transcriptional de-silencing in GRTS than GRH loci (Fig. 5b, c). In addition, GRTS and GRH loci did not show de-silencing at mRNA level in *mes*-*2* mutants by using a published microarray data [48] (Fig. 7d), nor in *met*-*2 set*-*25;set*-*32* mutants by mRNA-seq analysis in this study (Fig. 7d). In contrast, these targets were activated in *hrde*-*1* mutants (Fig. 7d). Taken together, these results suggest that transcriptional silencing at HRDE-1 targets is likely to be independent of H3K27me3 as well.

We note that, by performing H3K27me3 ChIP-seq in *met*-*2 set*-*25;set*-*32* mutant worms, we found no evidence of any H3K27me3-based compensating mechanism for the H3K9me3 loss—the triple HMT mutant worms did not show increased H3K27me3 in GRTS and GRH loci (Fig. 7b, c).

## Discussion

In plants, *S. pombe*, as well as *Drosophila,* nuclear RNAi-mediated H3K9me3 is essential to maintain the transcriptional silencing state at target loci. Here we show that H3K9me3 is completely dispensable for the maintenance of endo-siRNA-mediated transcriptional silencing at the whole-genome level in *C. elegans*. In addition, H3K9me3 is not required for exogenous dsRNA-triggered transcriptional silencing and heritable RNAi in a well-established native gene target. These findings shift the paradigm of the *C. elegans* nuclear RNAi pathway, a key model system of studying RNA-mediated transcriptional silencing and transgenerational epigenetics.

### The H3K9me3 contributions of MET-2, SET-25, and SET-32 in HRDE-1-dependent regions and elsewhere in the genome

*C. elegans* has several H3K9 HMTs. Before this study, it was unclear which of these HMTs are required for siRNA-mediated H3K9me3. Here, we provide experimental evidence supporting that germline nuclear RNAi-dependent H3K9me3 requires MET-2, SET-25, and SET-32.

Interestingly, the relative H3K9me3 contributions of these three HMTs are not the same at different targets. Their activities appear to be synergistic at the native targets (endo-siRNA-targeted), as each of the two mutant strains, *met*-*2 set*-*25* and *set*-*32,* showed >50% of H3K9me3 loss compared to the wild type. Such synergy may be the underlying mechanism that allows the high level of H3K9me3 at the native targets. The synergistic relationship is also evident for essentially all H3K9me3-enriched regions in the genome. The underlying mechanism for such apparent synergy is currently unknown. We also note that MET-2 SET-25 and SET-32 activities are not entirely mutually dependent, as the triple HMT mutant worms showed greater H3K9me3 loss than *met*-*2 set*-*25* or *set*-*32* mutants. For exogenous dsRNA-induced H3K9me3, MET-2 SET-25-dependent H3K9me3 and SET-32-dependent H3K9me3 are additive, suggesting that the two are independently triggered by dsRNA.

### RNAi-mediated transcriptional (or co-transcriptional) silencing at different types of targets

Our ChIP-seq analyses show that HRDE-1-dependent silencing at native targets (endo-siRNA targeted) leads to depletion of Pol II throughout the regions, suggesting that transcription initiation is prevented. In contrast, exogenous dsRNA-triggered RNAi does not significantly change the overall Pol II level at the target gene, but causes a shift of the Pol II profile instead (a reduction at the 3′ end and a gain in the gene body). This suggests that exogenous dsRNA-triggered RNAi does not block transcription initiation, but rather occurs during transcription elongation (also suggested previously by [19]) or co-transcriptionally. It is conceivable that the actual effect of RNAi on transcriptional silencing (and perhaps the degree of heritable silencing) is dependent on various features associated with the target gene (e.g., promoter, chromatin landscape, and siRNA).

### The role of H3K9me3 in *C. elegans* germline nuclear RNAi

Our findings are consistent with two models. Model 1: H3K9me3 is not involved in siRNA-mediated transcription silencing and its function lies in another not yet identified aspect of germline nuclear RNAi. Model 2: H3K9me3 is involved in siRNA-mediated transcription silencing, together with an H3K9me3-independent silencing mechanism. Although these two models are mutually exclusive at any given target locus, they may both occur in *C. elegans* and used by different loci/silencing mechanisms that involve siRNA. A previous study showed that SET-32 and SET-25 are required for HRDE-1-dependent silencing using a GFP transgene as the piRNA reporter or exogenous dsRNA-induced heritable RNAi reporter [21]. In our study, these HMTs are not required for heritable silencing at a native reporter gene, *oma*-*1.* Future studies are required to explain this difference. Transgene reporter is a powerful tool in studying RNAi. However, the efficacy of small RNA-mediated silencing on a transgene reporter appears to be dependent on a variety of factors, such as transgene structure [23], epigenetic history [49, 50], and DNA sequence [51]. On the other hand, detailed characterization of additional native targets is needed to address whether heritable RNAi is regulated in a target-specific, context-dependent manner.

## Conclusions

We found that three H3K9 HMTs, MET-2, SET-25, and SET-32, are required for germline nuclear RNAi-mediated H3K9me3 in *C. elegans.* Loss of the prominent H3K9me3 response in *met*-*2 set*-*25;set*-*32* mutant worms is not associated with any defect in germline nuclear RNAi-mediated transcriptional silencing at the native targets. Therefore, a high level of H3K9me3 is dispensable for the maintenance of the HRDE-1-dependent transcriptional silencing in *C. elegans* germline. In addition, we found that dsRNA-induced H3K9me3 is not required for transgenerational silencing at *oma*-*1*. We propose that transcriptional and heritable silencing of germline nuclear RNAi pathway in *C. elegans* can be maintained in an H3K9me3-independent manner. Our discovery that H3K9me3 can be decoupled from transcriptional silencing in *C. elegans* provides a unique opportunity to study any H3K9me3-independent silencing mechanism, particularly the direct biochemical effect of AGO-siRNA complex on transcription and co-transcriptional processes.

## Methods

### Strains

Bristol strain N2 was used as a standard wild-type strain. This study used the following mutations: Chr I: *set*-*32(ok1457);* Chr II*: set*-*13(ok2697);* Chr III*: set*-*25(ok5021), hrde*-*1(tm1200);* Chr IV*: set*-*21(ok2327), set*-*26(tm3526),* and *set*-*9(red8);* Chr V*: met*-*2(n4256).* The *set*-*9(red8)* mutation has a stop codon and a frame shift in the first exon, which was generated in this study using the CRISPR-cas9-mediated genome editing [52, 53]. Genotyping primers and other relevant sequences are listed in Additional file 9: Table S2. All strains were cultured at 19 °C.

### Worm grind preparation

Worms were cultured on NGM plates with OP50 *E.coli* as a food source [54]. Synchronized young adult hermaphrodite animals were obtained by first using the bleaching method to collect worm embryos, which were hatched in M9 buffer without food, and then L1 larvae were released onto NGM plates with OP50 *E. coli*. Young adult worms were ground by mortar and pestle in liquid nitrogen and stored at −80 °C. Worm grind from ~5000 young adult worms was used for each assay in this study.

### Multigenerational heritable RNAi

Heritable RNAi experiments were conducted as previously described [18]. *oma*-*1* and *gfp* RNAi trigger sequences are reported in Additional file 9: Table S2. For *oma*-*1* RNAi, single nucleotide mismatch at every 30 nt was used to distinguish the trigger sequence from the native *oma*-*1* gene. All animals at P0 (with dsRNA feeding) and F1, F2, F3 (without dsRNA feeding) generations were collected at young adult stage and ground in liquid nitrogen.

### H3K9me3 ChIP-qPCR

Worm grinds of P0 generation of *oma*-*1* RNAi and *gfp* RNAi samples at young adult stage were used for crosslinking, sonication and ChIP according to the protocol described in [27]. H3K9me3 ChIP from ~5000 worms per grind yield 5–10 ng of DNA. 1 ng of ChIP DNA was used per qPCR reaction. qPCR was set up using KAPA SYBR FAST Universal 2× PCR Master Mix (KAPA Biosystems) on a Mastercycler EP Realplex real-time PCR system (Eppendorf) according to the manufacturer’s instructions. qPCR primers are listed in Additional file 9: Table S2. Each sample was processed in triplicate. Reported values for the *oma*-*1* RNAi H3K9me3 fold change were calculated using ∆∆CT analysis.

### mRNA and pre-mRNA qRT-PCR

Worm grinds of P0 (RNAi+) and F1, F2, F3 (RNAi−) generations were used for total RNA extraction with Trizol reagent (Life Technologies), followed by DNase I (NEB) treatment.

mRNA reverse transcription (RT). 1 μg of total RNA was used for the first-strand cDNA synthesis with SuperScript III RT kit (Life Technologies) and oligo(dT)_20_ primer (to enrich for mRNA).

Pre-mRNA RT. 2 μg of total RNA was used for the first-strand cDNA synthesis with SuperScript III RT kit (Life Technologies) and random hexamer primer mix (to capture pre-mRNA).

qRT-PCR. qRT-PCR was performed using KAPA SYBR FAST Universal 2× PCR Master Mix (KAPA Biosystems) on a Mastercycler EP Realplex real-time PCR system (Eppendorf) according to the manufacturer’s instructions. qPCR primers are listed in Additional file 9: Table S2. Each sample was processed in triplicate. Reported values for the fold change of mRNA and pre-mRNA at *oma*-*1* gene were calculated using ∆∆CT analysis.

### High-throughput sequencing

Pre-mRNA-seq. Pre-mRNA library was prepared as described in [40]. Anti-RNA Pol II S2 (ab5095, Abcam) antibodies were used for Pol II IP, followed by RNA isolation and library preparation.

mRNA-seq. Worm grinds (~5000 young adult worms) were used for total RNA extraction with Trizol reagent (Life Technologies). mRNA was enriched using the Poly(A) Purist MAG kit (Life Technologies) according to the manufacturer’s instructions. 0.5–1 μg of mRNA was used for mRNA-seq library preparation as described in [40]. A mixture of four different 4-mer barcodes was used for the 5′-end ligation in both mRNA and pre-mRNA-seq as indicated Additional file 1: Table S1.

All libraries were sequenced using Illumina HiSeq 2500 platform, with 50-nt single-end run and dedicated index sequencing. Dedicated 6-mer indexes were used to de-multiplex DNA ChIP-seq and RNA-seq libraries for different samples.

Data availability: De-multiplexed raw sequencing data in fastq format for all libraries were deposited in NCBI (GEO accession number: GSE86517).

### Data analysis

Sequencing reads were aligned to *C. elegans* genome (WS190 version) by using Bowtie (0.12.7) [55]. Only perfect alignments were used for data analysis. If a read was aligned to N different loci, it was counted as 1/N. Normalization based on sequencing depth of each library was used for all data analysis. In Fig. 5a and Additional file 6: Fig. S3a, besides sequencing depth, we also used the median values of H3K9me3(−) regions for data normalization. Otherwise, the background H3K9me3 levels in HMT mutants are artificially higher than the WT one, due to a high degree of H3K9me3 loss in a large fraction of the genome in the HMT mutants (e.g., Fig. 4a). The three-region Venn diagram was generated using a web-based software (http://www.benfrederickson.com/venn-diagrams-with-d3.js/). Custom R and python scripts were used in this study.

Curves that indicate twofold or 1.5-fold changes (FDR < 0.05) in all scatter plots were calculated using a script from [56]. Welch two-sample t test was used to calculate all *p* values.

## Additional files

**Additional file 1: Table S1.** List of experiments, libraries, sequencing depth and barcodes used in this study.

**Additional file 2: Figure S1.** H3K9me3 ChIP-qPCR analysis of *oma*-*1* dsRNA-triggered H3K9me3 response in WT and various mutants.

**Additional file 3: Figure S2.** H3K9me3 levels are plotted as a function of position on all chromosomes for WT and HMT mutants (the panels for chromosome V are also shown in Fig. 4a). A moving average with a sliding window of 50 kb was used to plot H3K9me3 levels.

**Additional file 4: Table S3.** RPKM values of 1-kb windows for all ChIP-seq libraries used in this study. GRTS loci, GRH loci, SET-32-dependent H3K9me3, MET-2 SET-25-dependent H3K9me3, and MET-2 SET-25;SET-32-dependent H3K9me3 are indicated.

**Additional file 5: Figure S4.** Identification of H3K9me3 that is dependent on SET-32, MET-2 SET-25, or MET-2 SET-25;SET-32. (a) Whole-genome H3K9me3 scatter plots (1 kb resolution) that compare various HMT mutants with WT. (b) Venn diagram analysis between two biological repeats for H3K9me3 that is dependent on SET-32, MET-2 SET-25, or MET-2 SET-25;SET-32.

**Additional file 6: Figure S3.** Boxplot analysis for the ratios between mutant and WT for H3K9me3, Pol II, and pre-mRNA in different subsets of the genome: GRTS loci (191 kb), GRH loci (215 kb), top 5-percentile H3K9me3 regions in WT (H3K9me3(+) regions, 4775 kb), and bottom 5–25-percentile H3K9me3 regions in WT (H3K9me3(−) regions, 20,200 kb).

**Additional file 7: Figure S5.** Whole-genome scatter plots (1 kb resolution) that compare various mutant with WT for (a) H3K9me3 ChIP-seq, (b) Pol II ChIP-seq, and (c) pre-mRNA and (d) mRNA-seq (gene-based).

**Additional file 8: Figure S6.** DAPI staining of germline nuclei from WT and *met*-*2 set*-*25;set*-*32* mutant adult hermaphrodite. Scale bar: 10 µm.

**Additional file 9: Table S2.** Oligonucleotides and other sequences used in this study.

**Additional file 10: Table S4.** RPKM values of all annotated genes for all mRNA-seq libraries used in this study. GRTS loci, GRH loci, and H3K9me3-enriched genes are indicated.

**Additional file 11: Table S5.** RPKM values of 1-kb windows for all pre-mRNA-seq libraries used in this study. GRTS loci, GRH loci, SET-32-dependent H3K9me3, MET-2 SET-25-dependent H3K9me3, and MET-2 SET-25;SET-32-dependent H3K9me3 are indicated.

## Abbreviations

HMT: histone methyltransferases
ChIP-seq: chromatin immunoprecipitation—high-throughput sequencing
RNAi: RNA interference
Endo-siRNA: endogenous small interfering RNA
dsRNA: double-stranded RNA
GRH: germline nuclear RNAi-mediated heterochromatin
GRTS: germline nuclear RNAi-mediated transcriptional silencing
FDR: false discovery rate
